# Suicide-preventive compulsory admission is not a proportionate measure – time for clinicians to recognise the associated risks

**DOI:** 10.1007/s40592-024-00190-6

**Published:** 2024-04-13

**Authors:** Antoinette Lundahl

**Affiliations:** Stockholm Centre for Healthcare Ethics, Department of Learning, Informatics, Management and Ethics, Karolinska Institutet, Stockholm, Sweden

**Keywords:** Suicide prevention, Compulsory admission, Proportionality, Medical ethics

## Abstract

Suicide is considered a global public health issue and compulsory admission is a commonly used measure to prevent suicide. However, the practice has been criticised since several studies indicate that the measure lacks empirical support and may even increase suicide risk. This paper investigates whether the practice has enough empirical support to be considered proportionate. To that end, arguments supporting compulsory admission as a suicide-preventive measure for most suicidal patients are scrutinized. The ethical point of departure is that the expected benefits of compulsory admission should outweigh the potential harms of the measure to be proportionate and defensible. It is concluded that, for most suicidal patients, suicide-preventive compulsory admission cannot be presumed to be a proportionate measure. To be so, the expected medical benefits of the measure should be greater than the potential increase in suicide risk and other harms that compulsory admission could entail. Instead of using compulsory admission as a suicide-preventive measure, extra safety measures may be needed during and after compulsory admission to prevent the risk of hospitalisation-induced suicide.

## Background

### Suicide as a public health issue

Suicide is the fourth most common cause of death for people between the ages of 15–29 worldwide, causing around 700,000 deaths per year, and WHO recognizes suicide as a public health priority (World Health Organisation 2023).

### Compulsory admissions as a suicide preventive measure

When a patient has been assessed as suicidal, one common measure is to hospitalise the patient to prevent suicide and treat their mental disorder – compulsory admission if considered necessary. Not doing so may even lead to legal repercussions for the care provider, something that has been argued to fuel clinicians’ use of compulsory care (Wang & Colucci 2017). There is a lack of statistics on how many patients are compulsorily hospitalised due to suicidality, but one study of compulsory admissions in 40 European countries indicated that ‘danger to self’ was the motive for more than half of such admissions (Wasserman et al. 2020). It is also commonly acknowledged that compulsory admission is widely used as a suicide-preventive measure; an intervention that has legal support in many countries. In some countries, assessed danger to self or others is even a criterion for applying compulsory psychiatric hospitalisation (Wang & Colucci, 2017; Borecky et al. 2019). Also, the intervention is supported by clinical guidelines on how to manage suicidal patients, though the guidelines usually stress that the patient’s needs should be decisive in which intervention is most appropriate (NICE guideline number NG225 2022; The Swedish Board of Health and Welfare 2022; Borecky et al. 2019). However, this practice has been repeatedly criticised, not least because compulsory admission can entail negative effects on patients and the suicide-preventive effect has weak evidential support (Wang & Colucci 2017; Borecky et al. 2019).

### Need for ethical and legal proportionality when deciding on compulsory admission

When deciding on compulsory admission, the clinician should balance the pros and cons of such an intervention. From a medico-ethical point of view, it is widely agreed that healthcare should follow four main ethical principles: be beneficial to the patient, not harm the patient, respect the patient’s autonomy, and treat patients justly. These principles are known as Principlism. When different moral principles stand in conflict, e.g., when an infringement on autonomy can be both beneficial and harmful to the patient, the expected benefits must outweigh the potential harms of the intervention to be considered medically and ethically proportionate (Beauchamp et al. 2013).

The ethical deliberations above are reflected in The European Convention on Human Rights, which states that compulsory psychiatric interventions are an infringement on the individual’s fundamental rights and freedoms and that such infringements should not be arbitrary but strictly regulated by law and medically justified. According to the praxis of the European Court, coercive measures must also be proportionate, meaning that the coercive measures must serve a legitimate aim, that coercion be necessary to achieve this aim, and that the expected benefits must exceed or at least equal the potential harm from the coercion (European Convention on Human Rights Article 5 and 8; Åkerman et al. 2022; Nilsson 2023). Further, the UN Human Rights Committee, General Comment 35, states that any deprivation of liberty must be necessary and proportionate, used to protect the individual or others from serious harm, applied as a measure of last resort and for the shortest appropriate time, and restricted by law (UN Human Rights Committee 2014; Martin and Gurbai 2019).

### The aim of this paper

Notwithstanding the critique against the use of compulsory admission as a suicide preventive measure (Borecky et al. 2019; Wang & Colucci 2017), clinical practice does not seem to have changed so far. An indication of this is that compulsory admissions are increasing in many countries, and increased suicide risk is a common reason for such care (Lin et al. 2019; The Swedish Board of Health and Welfare Statistics 2023; Lee and Cohen 2021; Conlan-Trant et al. 2022).

In this paper, I look closer at the empirical support for and against using compulsory admission as a suicide-preventive measure and I conclude that it is not a proportionate intervention. Instead, I will argue that involuntary admissions are only proportionate if the medical benefits of treating the disorder are expected to be greater than the potentially increased suicide risk or other harms that are associated with compulsory hospitalisation. I will draw on the evidence and arguments presented in the debate of later years, in particular, by Large et al. (2017), Kapur et al. (2015), Borecky et al. (2019), Wang & Colucci (2017), Large & Kapur (2018), and Bryan (2021).

#### Arguments supporting the use of compulsory admission as a suicide-preventive measure

The arguments that have been used in both the academic and clinical debate on psychiatric admissions as a suicide preventive measure could be summarised as follows (Bryan 2021; Large & Kapur 2018; Borecky et al. 2019; Wang & Colucci 2017):

For most patients assessed with increased suicide risk, the benefits from psychiatric admission, including compulsory admission, exceed the potential harms because it prevents suicides more than no admission.

If there is evidential support for the argument above, then one can hold that compulsory admission is a proportionate measure for most people with increased suicide risk.

#### Analysis of the argument

I will begin by presenting the problem of selecting which patients are suicidal and, as a consequence, potential subjects to suicide-preventive compulsory admissions. If the accuracy of such a selection is low, then many patients risk being compulsorily admitted for the ‘wrong’ reason. That could further affect the proportionality of the measure.

Next, I will investigate the support for hospitalisation in general, including both voluntary and compulsory admissions, as a suicide-preventive measure. The reason is the larger amount of data available, which at least indicates in which way compulsory admissions can be associated with suicidality. Depending on national legislation and clinical traditions, the percentage of compulsory admissions, compared to all psychiatric admissions, varies between countries. For example, the rate of compulsory admissions constitutes around 15% of all psychiatric admissions in several European countries (with variations from 3 to 30%), and up to 54% in the US (Salize & Dressing 2004, Lay et al. 2012, Lutterman et al. 2017). Another reason is that voluntary care could be considered the main alternative to involuntary care when a patient is assessed as suicidal, and therefore it is clinically relevant to look at the effect of all psychiatric admissions.

Lastly, I will present the support for compulsory admission, separated from voluntary admissions, as a suicide-preventive measure.

### The selection problem

To discern which patients are suicidal in the first place, psychiatrists usually assess suicide risk in some form. However, suicide risk assessments are not good tools for screening for suicidality. They have limited sensitivity (missing around half of the patients who will commit suicide), limited specificity, and a low short-term positive predictive value (PPV), meaning that only a small portion of patients identified as high-risk will die by suicide. The short-time PPV for one of the better assessment tools today (the Columbia-Suicide Severity Rating Scale) indicates that out of 500 patients assessed with high suicide risk, only one will commit suicide (Bjureberg et al. 2022, Franklin et al. 2017, Large 2018). This presents a problem with selection; if patients are selected for compulsory admission based on suicide risk, more than 99% will be hospitalised against their will even though they will not commit suicide in the near future (and 95% will never). Therefore, it has been advised against letting suicide risk assessments guide management or using their outcome as a motive for liberty-infringing interventions (Bjureberg et al. 2022; Large 2018; Wang & Colucci 2017; NICE guideline number NG225 2022).

### Data indicating that hospitalisation fails to prevent suicides and may increase suicide risk

A large meta-analysis with pooled data worldwide from the last 60 years has shown an association between hospitalisation (in general) and suicide that is stronger than the association between smoking and lung cancer. Prior psychiatric hospitalisation is the strongest known risk factor for suicide – far stronger than all psychiatric diagnoses taken together (Walsh et al. 2015; Large & Ryan 2014; Franklin et al. 2017). There are no controlled studies on the relationship between hospitalisation and suicide, probably because it would be difficult to receive ethical approval, and therefore one cannot infer causation. However, based on several studies and meta-analyses, it has been argued that it is likely that hospitalisation in and of itself could entail increased suicide risk, even though a large part of hospitalisation-related suicides are thought to be explained by selection (Large et al. 2017; Large & Kapur 2018).

To support this claim of possible causation between hospitalisation and suicide, researchers have referred to statistician and epidemiologist Austin Bradford Hill’s criteria for assessing a possible causal association between an environmental factor and a disease. These criteria can be helpful in situations when controlled studies are difficult to obtain, as in the case at hand (Large et al. 2017). In short, following Hill’s criteria, several phenomena can indicate causality: the strong association between hospitalisation and suicide, which has been observed by different researchers in different locations over a long period, the positive correlation between the amount of hospital care (long stays or many admissions) and suicide, the weak association with the patient’s suicidality at admission, patients’ accounts of traumatising experiences during admission (e.g., feelings of hopelessness and loss of control, which could partly explain the possible causality), and the significant variation in suicide numbers between clinics. The suicide risk is extra high immediately after admission and discharge and remains elevated for years (Chiles et al. 2018; Bjureberg et al. 2022; Large et al. 2017; Large & Kapur 2018; Walsh et al. 2015; Priebe 2019; Large & Ryan 2014; Hunt et al. 2013; Chung et al. 2019).

Other sources have argued against this suggested causal relationship between hospitalisation and suicide, holding that the strong association between hospitalisation and suicide is mainly explained by patient selection. Those suicides that are still related to inpatient care, have been attributed to a lack of safety measures at the ward, e.g. removal of ligature points, and an unwelcoming environment. In short, the argument goes that it does not seem plausible that hospitalisation does more harm than good, though hospitalisation sometimes fails to prevent suicide (Kapur et al. 2015; Large & Kapur 2018).

### Data indicating that hospitalisation may have suicide-preventive effects

There are no controlled studies that show that hospitalisation prevents suicides but, as mentioned above, controlled studies on this subject are difficult to perform. There are, however, some studies that have suggested suicide-preventive effects of hospitalisation, at least for certain subgroups of patients.

One study compared suicide data from inpatient care with home treatment services in the UK and found indications that inpatient care could be better at preventing suicides. However, the study had methodological problems that made direct comparisons difficult, and therefore it has been argued that no such conclusion can be drawn (Hunt et al. 2014; Becker & Rüsch 2014; Kapur & Large 2018).

A 10-year observational study on clinical management of patients who had self-harmed indicated that psychiatric hospital admission of people over the age of 65 might be associated with lower suicide risk compared with those in the same age group who were not admitted, but the numbers were small and the CIs overlapped. Hospital admission was not associated with decreased suicide risk for any other age group in the study. On the whole, hospital admission was associated with the highest risk for suicide, but the authors interpreted this as a selection effect (Kapur et al. 2015). Considering that only a smaller portion of inpatients in psychiatry are over the age of 65 (around 5% in Sweden) and that most inpatients are between 24 and 44 years old, the results of this study do not support the argument that hospitalisation is suicide-preventive for most patients (Thompson et al. 2004; Wieselgren 2010). When it comes to compulsory admission specifically, as will be discussed in the next section, most involuntarily hospitalised patients are also under the age of 45 (Audini and Lelliott 2002, The Swedish Board of Health and Welfare Statistics 2022).

### Summary concerning psychiatric admission in general and suicidality

In summary, there are no studies that manage to show that psychiatric admission prevents suicides more than no admission; if anything, the studies indicate that hospitalisation fails to prevent suicides. There is a strong association between hospitalisation and suicide, and statistical analysis of the data suggests that this association can not only be explained by patient selection. Instead, the data indicates that hospitalisation may causally increase the risk for suicide both short-term and long-term, at least to a smaller extent (Walsh et al. 2015; Large & Ryan 2014; Franklin et al. 2017, Large et al. 2017, Large & Kapur 2018).

### The beneficence of compulsory treatment

When looking at compulsory admissions, it is important to also take into account the potential benefits of such care. Several severe mental disorders can be effectively treated in the hospital, e.g., manic psychoses, acute psychotic states, psychotic depressions, etc. In these cases, the patients are likely to lack decision capacity and insight into their condition, and therefore it can be difficult or even impossible to provide them with necessary treatment if treated voluntarily. For these patients, effective treatment could strongly improve their quality of life and, as a consequence, potentially reduce their suicide risk (NICE n.d.; Owen 2008, D’Anci et al. 2019).

A little more than half of compulsorily admitted inpatients suffer from psychotic or manic states (Lay et al. 2019; The Swedish Board of Health and Welfare Statistics 2022). On the other hand, based on clinical experience and the profile of these diagnoses, patients with manic or psychotic disorders are rarely detained because they are assessed as suicidal, but to treat the severe condition (NICE n.d.; DSM V 2013).

For patients with less disabling conditions, the beneficence of compulsory hospital treatment depends on the patient, the condition, and the severity of the disorder (NICE n.d.).

### Compulsory admissions and suicidality

Suicide risk is increased in all places where people are held involuntarily, e.g., hospitals and prisons (NICE guideline number NG225 2022; Chiles et al., 2018; Large et al. 2017). Case-control studies on inpatient suicides (which is a small fraction of all suicides) during compulsory admission have shown conflicting results, which could be explained by methodological differences and the limited number of inpatient suicides, e.g., one study with diagnosis-matched controls indicated compulsory admission as an independent risk factor for inpatient suicide, while another study without diagnosis-matching indicated that compulsorily admitted patients had less risk of inpatient suicide (Williams & Colucci 2018; King et al. 2001; Hunt et al. 2007). On the other hand, general liberty-infringing measures such as locked doors, constant monitoring, and removal of potentially harmful objects have not been shown to prevent inpatients from harming themselves and some of these interventions even seem to increase self-harm (James et al. 2012; Bowers et al. 2008). A 15-year observational study compared matched patients admitted to locked or open wards, respectively, and found that locked wards were associated with a higher risk of suicide attempts and absconding than open wards and locked wards did not seem to prevent suicides better than open wards (Huber et al. 2016). Another study has shown that patients who perceived coercion during their hospital stay were more likely to commit suicide attempts after discharge than patients who did not experience coercion (Jordan et al. 2020). These findings suggest that the removal of a patient’s autonomy during hospitalisation may have suicidogenic properties.

The claim of compulsory admission having suicidogenic properties is perhaps most supported by studies done on patients with recurrent self-harm behaviour (self-harm with or without suicidal intent), e.g. patients with borderline personality disorder, who are overrepresented in compulsory care (Bender et al. 2001; Office for Health Improvement and Disparities 2023; Paris 2019; Linehan 1993; NICE guideline number NG225 2022; NICE guideline number 78 2009). Removing agency from these patients, often young women, by ordering compulsory admission, restricting their freedom, or making decisions for them seems to perpetuate and reinforce the patients’ suicidality (Linehan 1993; Coyle et al. 2018; Chiles et al., 2018; NICE guideline number 78 2009; Lundahl et al. 2023; Bowers et al. 2008).

Nevertheless, the negative effects of involuntary hospitalisation do not seem to be confined to patients with self-harm behaviour. As has been summarised by Borecky et al. (2019), several studies indicate that involuntary admission is associated with negative thoughts and feelings, such as loss of self-control, helplessness, shame, increased preoccupation with suicidal thoughts in response to feeling controlled, etc. It has also been shown that many suicidal patients benefit from increased self-efficacy, learning skills on how to handle crises and difficult emotions on their own, and increased autonomy – the opposite of what is associated with involuntary admissions (Bryan 2021; Borecky et al. 2019; NICE guideline number 78 2009; Chiles et al., 2018). As of yet, no studies indicate that compulsory admission would decrease overall suicide risk.

Another point in this argument is that compulsory admission is an infringement on the person’s autonomy, which could be compared in some ways to imprisonment, even if done in the patient’s best interest. This is a harmful act in and of itself, which should be considered when weighing up the potential benefits and harms of the measure.

### Summary concerning psychiatric admission in general and suicidality

The current evidence indicates that compulsory admissions have more potential to increase suicidality than psychiatric admissions in general. There are also other harms attached to compulsory admissions, including both negative therapeutical effects and the harm of depriving a person of their autonomy.

### Conclusion of the analysis

When summoning up the evidence, empirics do not support the argument presented. Neither psychiatric admission in general, nor compulsory admission, has been shown to reduce suicide risk. Rather, it seems that hospitalisation either fails to prevent suicide or has the potential to increase the suicide risk – and compulsory admissions even more so. Compulsory admissions also have more potential to cause other types of harm, as mentioned above. Hence, given the current state of evidence, for most people with increased suicide risk, it is not a proportionate measure to admit them involuntarily if the main purpose is to prevent suicide. To make compulsory admission a proportionate measure, the expected benefits of the medical treatment should outweigh the potential increase in suicide risk, or other harms, that the measure can entail.

## Discussion

### Compulsory admissions and the precautionary principle

In this line of argument, one can refer to the Precautionary principle, which says that measures against a possible hazard should be taken even if the existence of such a hazard lacks solid scientific support (Sandin 2004; Munthe 2020). There is no absolute proof of causality between compulsory admissions and suicide or other harms (as those mentioned above). However, there is enough evidence to say that compulsory admissions are likely to entail such negative effects to some extent, and therefore I argue it is reasonable to take preventive measures – in this case, to use compulsory admissions with caution and awareness of the potential risks attached.

### The current use of compulsory admission as a suicide-preventive measure

In clinical reality, little if nothing has changed, as previously argued. Maybe because clinicians do not know what else to do with a suicidal patient (there could be a lack of outpatient treatment alternatives) and, intuitively, admitting them to the hospital – even involuntarily – seems like the safest thing to do. Also, not forcibly admitting a suicidal patient who later commits suicide can lead to medicolegal repercussions - something that has been argued to increase the use of compulsory admissions (Borecky et al. 2019; Lundahl et al. 2018). Another explanation is that hospitalisation of suicidal patients has been the traditional management for a long time and there could be resistance to changing established routines even if evidence indicates that the routines should be changed – a phenomenon known as path-dependency (Pierson 2000). Further, there seems to be a generally accepted belief, both among psychiatrists and in the community, that hospital admission can prevent suicides (Priebe 2019). This ‘priming’ of people’s beliefs, i.e., the preformed attitude that affects how they interpret information (Kahneman 2011), could be one of the reasons why the critique against compulsory admissions has been disregarded to a large extent.

### The selection problem

The problem with the selection of suicidal patients is another issue I have addressed in this paper. Even if suicide risk assessments are not considered to be clinically relevant, they still seem to be used extensively, even mandatorily, in several countries – albeit under different names – and are used as both predictive tools and to determine management decisions, including coercive measures (Large 2018) Graney et al. 2020; The Swedish Board of Health and Welfare 2023; Ryan & Maria 2020). Recently, the UK has advised against using suicide risk assessments as a tool for making predictions or determining care management in isolation from other factors (NICE guideline number NG225 2022). Yet, to assess the patient’s needs of safety, UK clinicians are demanded to make ‘risk formulations’, which, by the looks of it, contain the same balancing of suicide protective and aggravating factors that are normally included in a suicide risk assessment (NICE guideline number NG225 2022). It is not a farfetched guess that, in clinical practice, such ‘risk formulations’ will be used for selecting patients for suicide-preventive compulsory admissions.

### What can be done instead of compulsorily admitting a suicidal patient?

If compulsory admission is not an adequate measure for suicide prevention, then the clinician may ask what to do with the suicidal patient instead.

So far, this area has not been thoroughly researched – perhaps because many clinicians have settled on using psychiatric admission as the standard way of managing suicidal patients. Still, there are some studies done in recent years, showing that various community treatments could be a way of decreasing the use of compulsory admissions (Lay et al. 2018; Aagaard et al. 2017). Such treatments include, for example, psycho-education for learning self-management skills, regular contact with a mental health worker, and crisis planning (Lay et al. 2018). In general, learning the patient how to handle negative emotions and consequent suicidal thoughts constructively, without admitting the patient, seems to have suicide-preventive effects – at least for some patients (Coyle et al. 2018; Chiles et al., 2018; Bryan 2021). Another option for the clinician is the use of Crisis Resolution Teams, which can make home visits and be used as an alternative to hospital admission – however, the results of that intervention have been varying (Wheeler et al. 2015). Self-referred brief voluntary admissions could be considered since it has been shown to reduce the use of compulsory admission for some patients (Westling et al. 2019).

Recognising the problem of using compulsory admission as a suicide-preventive measure could maybe prompt more research on what type of clinical management is more effective. That would be desirable. Likewise, to raise mental health workers’ awareness about the risks associated with compulsory admissions, for example through education.

For patients who need to be compulsorily admitted, for example, to treat a melancholic depression or a psychotic state, it seems likely that a welcoming and calm environment in the ward could decrease feelings of hopelessness and distress. Clinicians should be aware of the possibly increased suicide risk associated with compulsory admission and take necessary precautions. The latter, one may speculate, could consist of carefully observing the patient, offering the patient different types of support to cope with the situation, and making sure that the patient gets an outpatient contact that could follow up after discharge. Another precaution could be not to use more coercive measures or longer compulsory admissions than necessary to treat the severe mental disorder.

## Summary and closing remarks

In this paper, the current practice of using compulsory admission as a suicide preventive measure is challenged. The selection of suicidal patients is troubling to begin with, considering the low predictive value of suicide risk assessments; most people assessed to have a high suicide risk, and thus be eligible for suicide-preventive compulsory admissions, will not commit suicide. Therefore, many patients will be held in the hospital against their will even if they will never commit suicide (Wang & Colucci 2017; Large 2018; Borecky et al. 2019). No studies have been able to show that hospitalisation, voluntary or involuntary, reduces suicide risk and there is an increasing body of evidence showing that hospitalisation, and especially involuntary admission, is associated with an increased suicide risk that may be partially caused by the hospitalisation itself (Large et al. 2017; Large & Kapur 2018). If weighing up the evidence, compulsory admission as a suicide-preventive measure is not a proportionate measure from either a legal or medico-ethical perspective. For the care to be proportionate, the expected benefits of the medical treatment given during compulsory admission should outweigh the potential increase in suicide risk or other harms related to such care. Such harms include the moral cost of depriving people of their autonomy.

Finally, considering that compulsory admission may increase suicide risk in and of itself, patients who get involuntarily admitted could require extra safety measures, both during admission and some time after discharge, and their disorder should be treated as effectively as possible to minimize the time of compulsory hospitalisation.
